# Evaluation of a campaign to reduce the catheter associated urinary infection

**DOI:** 10.1186/2047-2994-4-S1-P216

**Published:** 2015-06-16

**Authors:** F Vieira, I Devesa, D Peres, I Neves

**Affiliations:** 1 Infection Control and Antimicrobial Resistance Unit, Unidade Local de Saúde de Matosinhos, Matosinhos, Portugal; 2 Infectious Diseases Unit, Unidade Local de Saúde de Matosinhos, Matosinhos, Portugal

## Introduction

Like the majority of nosocomial infections, catheter associated urinary infection (CAUTI) causes an increase in morbidity, mortality, costs and antibiotics consumption.

## Objectives

To analyze the impact of a campaign to promote good practices in patients with urinary catheter in a 120 bed medicine department.

## Methods

Before the implementation of the campaign it was evaluated the urinary catheter rate and the CAUTI rate, as well as maintenance practices. Campaign implementation: during a week it was distributed different information daily with reminders to professionals sent by e-mail and flyer distribution focusing on the following information: urinary catheter indications; aseptic technique in catheter introduction; correct technique to empty collector bag and standard guidelines in patients with urinary catheter. Visits were carried out to the different units to promote the good practices. At the end of the week a open lecture was done to all professionals. Evaluation of the impact of the campaign was done by audits three and six months after.

## Results

The urinary catheter rate was 25.8%, 16,6% and 20%, before campaign, 3 and 6 months after, respectively. The CAUTI rate was 61.5%, 35% and 58.3%, before campaign, 3 and 6 months after, respectively. Audits showed errors in catheter maintenance, namely deficient perineal hygiene and insufficient decontamination of the collector bag tap.

## Conclusion

The implementation of this campaign contributed to reduce the urinary catheter rate and CAUTI rate. It also allowed correcting inappropriate practices.

## Disclosure of interest

None declared.

